# Targeting the IL-15/CD122 signaling pathway: reversing TRM cell-mediated immune memory in vitiligo

**DOI:** 10.3389/fimmu.2025.1639732

**Published:** 2025-07-28

**Authors:** Xiangxi Su, Fang Liu

**Affiliations:** Department of Dermatology, Beijing Chaoyang Hospital, Capital Medical University, Beijing, China

**Keywords:** vitiligo, tissue-resident memory T cell, IL-15, CD122, ruxolitinib, AMG 714

## Abstract

Vitiligo is a chronic autoimmune disorder in which melanocyte−specific CD8^+^ T cells destroy pigment−forming cells, producing persistent depigmented macules. Recurrence after treatment implicates tissue−resident memory T (TRM) cells that are maintained by interleukin−15 (IL−15) signaling. Here we review current insights into TRM−cell biology, summarize experimental and emerging clinical data targeting the IL−15/CD122 axis—including the ongoing Phase 2a AMG 714 trial—and discuss combination strategies with approved topical Janus kinase inhibitors such as ruxolitinib cream. Disrupting IL−15 may offer durable repigmentation with minimal systemic immunosuppression.

## 1 Introduction

Vitiligo, affecting 0.5–2% of the global population, is a recurrent depigmenting disorder with profound psychosocial impacts (1). Despite transient efficacy of topical corticosteroids and phototherapy, relapse rates exceed 40–50% within 2–15 months post-treatment, often involving both previous and novel sites (2). This recurrence pattern implicates tissue-resident memory T cells —long-lived immune sentinels maintained via IL-15 signaling—as key mediators of autoimmune memory (3). Recent advances in TRM biology and IL-15 pathway modulation offer transformative potential for vitiligo management, warranting a systematic evaluation of therapeutic targeting strategies.

## 2 Functional roles of TRM cells in vitiligo

### 2.1 Phenotypic characteristics and cutaneous localization

The etiology and pathogenesis of vitiligo have not been fully elucidated. It is widely accepted that the disease results from the combined effects of genetic, immune, oxidative stress, and environmental factors, leading to melanocyte destruction or impaired melanin synthesis (4). Both lesional and non-lesional skin in vitiligo patients harbor melanocyte-specific CD8+ TRM cells, which predominantly localize to depigmented areas (5). This observation underscores the close relationship between melanocyte destruction and the immune-mediated actions of CD8+ TRM cells.

These cells, marked by CD69, CD103, and CD49a expression, persist in the epidermis and basement membrane, surveilling tissues under physiological conditions while promoting autoimmunity in disease states (6, 7). These adhesion molecules which upon down regulation of KLF-2 (Kruppel like factor) and low level expression of CCR7, suppresses S1P1 (sphingosine-1-phosphate receptor1) activity and further allows TRMs to remain in peripheral tissues (8).

### 2.2 Molecular mechanisms of TRM cell-mediated melanocyte destruction

In vitiligo, CD8+ TRM cells are capable of expressing TNF-α, IFN-γ, as well as other chemokines and cytotoxic factors, directly inducing apoptosis and inhibiting melanogenesis (3). CD8+ TRM cells attack melanocytes (MCs), and the IFN-γ they produce stimulates keratinocytes to express CXCR3. CXCR3 binds to CXCL9 and CXCL10, recruiting additional MC-reactive T cells and perpetuating existing vitiligo lesions through a self-reinforcing “IFN-γ–CXCL axis.” (9, 10) Single-cell RNA sequencing reveals that lesional TRM cells exhibit a twofold increase in IFN-γ production compared to healthy skin, highlighting their role as a primary source of cytokines and their functional significance. Besides, TRM cells in non-lesional skin exhibit an intermediate activation state with upregulated IFN-γ responsiveness, which may explain the potential for vitiligo recurrence at distant sites (11).

## 3 Interleukin-15 signaling pathway: a key regulator of TRM cell survival and function

### 3.1 Molecular composition of the IL-15/CD122 signaling pathway

The interleukin-15 (IL-15) signaling pathway plays a critical role in regulating the development, maintenance, and function of tissue-resident memory T (TRM) cells. The IL-15 receptor exhibits a complex architecture and can exist in multiple configurations, including monomeric, heterodimeric, and heterotrimeric forms. Among them, the heterotrimeric IL-15 receptor is composed of three subunits: IL-15Rα (CD215), IL-2/15Rβ (CD122), and the common γ-chain (CD132). The heterodimeric form contains CD122 and CD132 but lacks CD215. Importantly, IL-15 can be presented in trans, where IL-15 bound to CD215 on one cell (e.g., a keratinocyte) is presented to neighboring cells expressing CD122/CD132 (e.g., TRM cells), facilitating effective signal transduction (12).

Experimental evidence from murine models and *in vitro* cultures has demonstrated that melanocyte-specific CD8^+^ TRM cells express the IL-2/15Rβ chain (CD122), while keratinocytes are the primary source of IL-15 and express CD215. This cellular arrangement enables keratinocytes to support TRM cell survival and effector function by trans-presenting IL-15, reinforcing their pathogenic role in autoimmune diseases such as vitiligo (7, 13).

### 3.2 Experimental evidence for IL-15 in promoting TRM cell function

Animal studies have shown that IL-15-deficient mice exhibit impaired TRM cell formation and maintenance, while IL-15 enhances TRM cell function *in-vitro*, highlighting the cytokine’s essential function in TRM cell homeostasis. *In vitro*, IL-15 stimulation enhances TRM cell survival, proliferation, and cytotoxic activity (14, 15).

**Figure 1:**
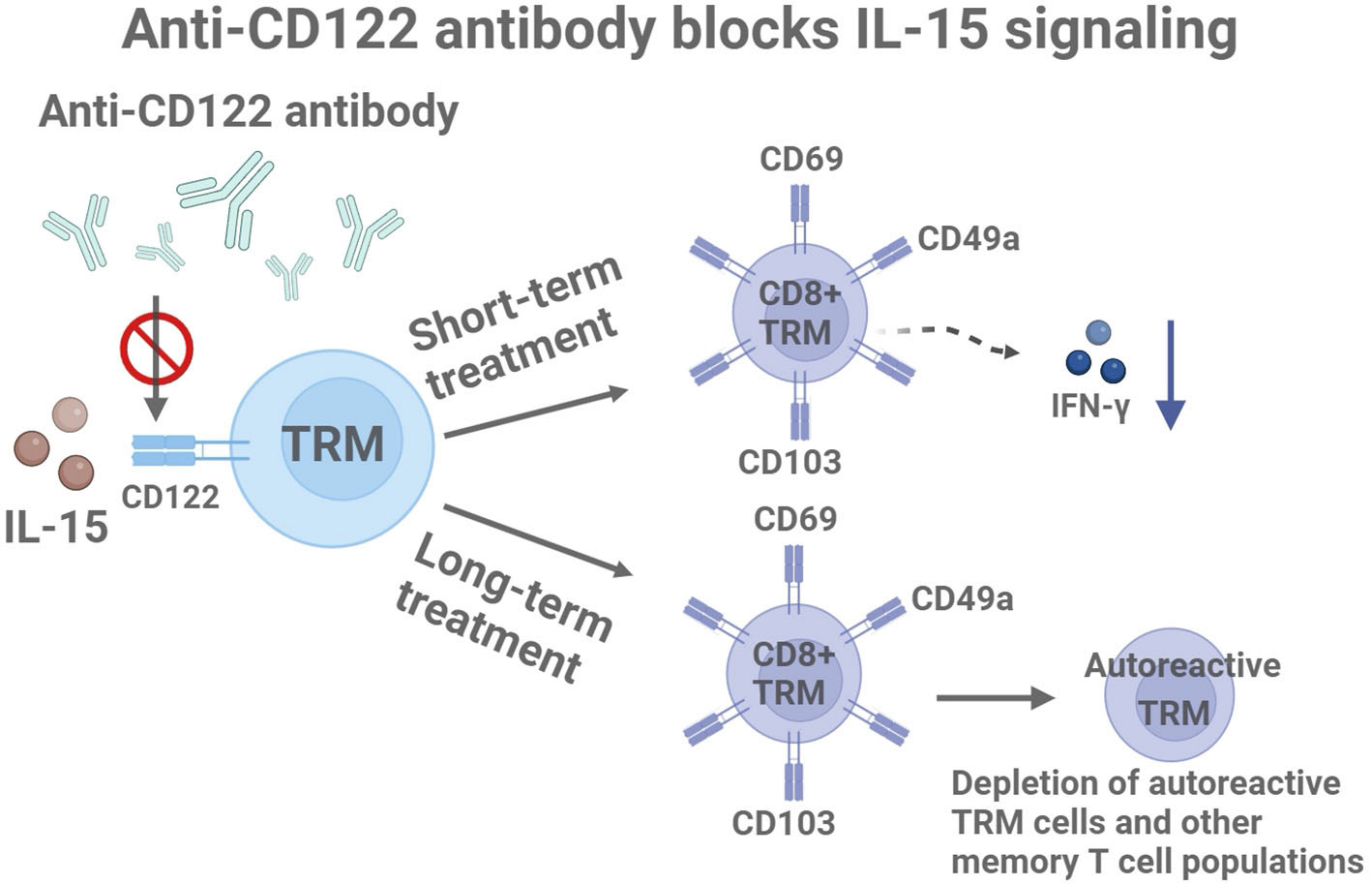
Molecular mechanisms of anti-CD122 antibodies in the treatment of vitiligo: short-term effects and long-term drug delivery. Created with BioRender.com.

In the context of vitiligo, IL-15 has been shown to drive the activation of CD49a^+^CD103^+^CD8^+^ TRM cells enriched in lesional skin. Upon IL-15 stimulation, these TRM cells rapidly upregulate effector molecules such as granzyme B and perforin, leading to targeted destruction of melanocytes. Furthermore, IL-15-induced IFN-γ production from TRM cells contributes to local inflammation and disease persistence (16–18) (**Figure 1**).

### 3.3 Role of the JAK-STAT pathway in IL-15 signaling

The downstream signaling cascade initiated by IL-15 is predominantly mediated through the Janus kinase (JAK)-signal transducer and activator of transcription (STAT) pathway. Upon IL-15 binding to its receptor complex (CD122/CD132), associated kinases JAK1 and JAK3 become activated. This activation leads to the phosphorylation and subsequent dimerization of STAT5, which translocates to the nucleus to modulate gene expression critical for TRM cell survival and cytotoxicity (19, 20).

The JAK-STAT pathway thus serves as a key conduit through which IL-15 exerts its biological effects on TRM cells. Inhibition of JAK kinases has been shown to disrupt IL-15 signaling, reduce TRM cell activity, and alleviate tissue damage in autoimmune conditions, supporting the therapeutic potential of JAK inhibitors in treating diseases like vitiligo.

Together, these findings underscore the pivotal role of IL-15 and its downstream JAK-STAT signaling axis in regulating TRM cell function and maintaining autoimmune responses in vitiligo.

## 4 Therapeutic strategies targeting the IL-15/CD122 pathway

### 4.1 Preclinical studies of anti-CD122 antibodies

The blockade of CD122, a subunit of the IL-15 receptor, has been tested in a vitiligo mouse model using specific monoclonal antibodies. Data indicate that short-term treatment reduces the effector function of TRM cells by decreasing IFN-γ production, resulting in significant repigmentation in treated mice. Prolonged systemic administration over 8 weeks leads to the depletion of autoreactive TRM cells and other memory T cell populations (7, 21) (**Figure 1**). These findings suggest that targeting IL-15 signaling through CD122 may be an effective strategy for treating vitiligo and achieving durable disease reversal.

### 4.2 Preclinical studies of anti- IL-15 antibodies and combination therapies

**Figure 2:**
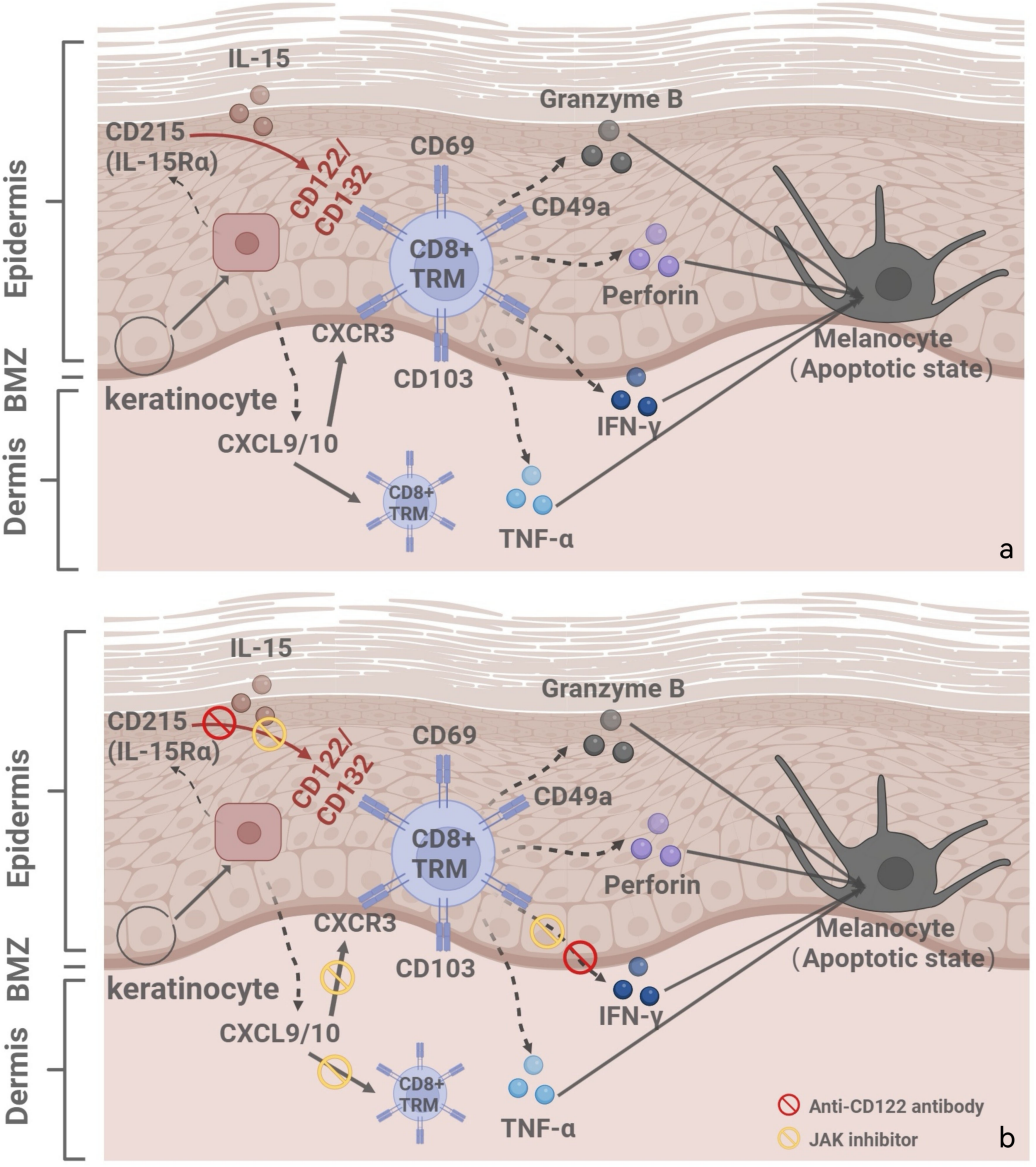
**(a)** Illustrates the vitiligo pathogenesis and recurrence mechanisms: IL-15/CD122 signaling pathway, IFN-γ-chemokine axis, with its associated positive-feedback loop. **(b)** Combination treatment strategies for vitiligo: anti-CD122 antibodies and JAK inhibitors. Created with BioRender.com.

The dual role of IL-15 in immune homeostasis may increase therapeutic complexity. Currently, IL-15 antagonizing antibodies (e.g., AMG 714) have demonstrated efficacy in treating autoimmune diseases such as celiac disease and ankylosing spondylitis (22), supporting their potential application in vitiligo. A Phase 2 clinical trial (NCT04338581), sponsored by the U.S. National Institute of Allergy and Infectious Diseases, is currently evaluating AMG 714 in adults with vitiligo. As of 2025, no peer-reviewed clinical outcomes have been published, and results are still pending. Future research should also explore combination therapies for vitiligo, such as co-administration with JAK inhibitors (e.g., ruxolitinib), which may synergistically suppress IFN-γ signaling and enhance therapeutic efficacy (23) (**Figure 2**). While targeting IL-15/CD122 signaling offers therapeutic promise, it is essential to consider the broader immunological role of IL-15. This cytokine supports the homeostasis of NK cells and memory CD8^+^ T cells, which are crucial for antiviral and antitumor immunity (24). Therefore, systemic IL-15 blockade may carry risks of immunosuppression. Future strategies should aim to minimize such risks, for example, through localized delivery or intermittent dosing schedules that preserve systemic immune integrity.

## 5 Future directions and conclusions

The IL-15/CD122 signaling axis has emerged as a promising therapeutic target in vitiligo due to its critical role in the maintenance and effector function of tissue-resident memory T (TRM) cells. Although current research is largely limited to preclinical studies and animal models, the encouraging outcomes in other autoimmune diseases—such as rheumatoid arthritis and cancer—highlight the translational potential of IL-15-targeted strategies (25). In murine models of vitiligo, blockade of IL-15 or its receptor subunits, particularly CD122, leads to reduced TRM cell activity, suppression of IFN-γ signaling, and sustained repigmentation. These findings underscore the pathway’s potential to interrupt autoimmune memory and promote long-term disease control. To facilitate the clinical translation of IL-15/CD122 pathway-targeted strategies in vitiligo, future research should advance along multiple directions. To begin with, understanding the phenotypic and functional heterogeneity of TRM cells is particularly critical, as distinct subsets may vary in their sensitivity to IL-15 blockade and their capacity to mediate melanocyte destruction. For example, CD49a^+^ TRM cells—co-expressing CD103—are enriched in lesional vitiligo skin and display enhanced cytotoxicity, granzyme B/perforin production, and stronger IL-15 responsiveness. In contrast, CD49a^-^ subsets may serve more homeostatic or regulatory roles. Therefore, therapeutic responses to IL-15 blockade may vary depending on the dominant TRM subset in a given lesion. This suggests the potential for subset-specific sensitivity, and indicates that patients with CD49a^+^-dominant TRM infiltrates may benefit more from IL-15–targeted interventions (26, 27). Recognizing these differences may influence therapeutic responses and recurrence risk, providing a rationale for precision-targeted interventions. In addition, the mechanisms underlying the interactions between TRM cells and other immune components—such as regulatory T cells (Tregs) and dendritic cells (DCs)—remain to be fully elucidated. For instance, type 1 regulatory T cells (Tregs) can promote the generation of CD8^+^ TRM cells by upregulating TGF-β and CD103, suggesting the existence of a synergistic regulatory axis (28, 29). Conversely, Tregs may also exert antagonistic effects—by limiting IL-15 trans-presentation by CD11b^+^ dendritic cells, they suppress the expansion of memory T cells, including TRM-like subsets (30). Notably, the development of TRM cells is further influenced by TGF-β signals derived from either Tregs or keratinocytes, which drive the expression of CD103 and CD69 and may interact with IL-15–trans-presenting dendritic cells (31). From a clinical perspective, rigorously designed trials are required to evaluate the safety, dosage, and long-term efficacy of anti-CD122 antibodies and IL-15 antagonists. The combination of IL-15 blockade with established immunomodulatory therapies, such as JAK inhibitors, holds promise for enhancing repigmentation outcomes and minimizing relapse, warranting further mechanistic and clinical validation. Given the immunological functions of IL-15 in systemic immunity, localized administration strategies—such as topical anti-CD122 formulations or intralesional delivery—may help mitigate off-target effects. These approaches can concentrate drug action within lesional skin while preserving peripheral immune surveillance, thus minimizing risks of opportunistic infections or tumor progression.

In conclusion, targeting the IL-15/CD122 signaling pathway represents a novel and mechanistically grounded approach to disrupting autoimmune memory in vitiligo. With continued research, this strategy may offer durable repigmentation and sustained remission for patients with vitiligo.
